# Injuries, Sequelae, and Treatment of Lightning-Induced Injuries: 10 Years of Experience at a Swiss Trauma Center

**DOI:** 10.1155/2012/167698

**Published:** 2012-05-13

**Authors:** Carmen A. Pfortmueller, Yang Yikun, Monika Haberkern, Erwin Wuest, Heinz Zimmermann, Aristomenis K. Exadaktylos

**Affiliations:** ^1^University Department of General Internal Medicine, Inselspital Bern, 3010 Bern, Switzerland; ^2^University Department of Emergency Medicine, Inselspital Bern, 3010 Bern, Switzerland; ^3^Swiss Federal Statistical Office, 2000 Neuchâtel, Switzerland

## Abstract

*Principals*. Lightning is one of the most powerful and spectacular natural phenomena. Lightning strikes to humans are uncommon but can cause devastating injuries. We analyzed lightning-related admissions to our emergency department from January 2000 to December 2010 to review and highlight the main features of lightning-related injuries. *Methods*. All data were collected prospectively and entered in the emergency department' database (Qualicare Switzerland) and retrospectively analyzed. *Results*. Nine patients with lightning-related injuries presented to our emergency department. Four were female, and five were male. The most common site of injury was the nervous system (6 out of 9 patients) followed by the cardiovascular system (5 out of 9 patients). The third most common injuries occurred to the skin (3 out of 9 patients). Four of the patients had to be hospitalized for further observation. *Conclusion*. Reports of lightning strikes and related injuries are scarce. The establishment of an international register would therefore benefit the understanding of their injury patterns and facilitate specific treatment.

## 1. Introduction

Lightning is one of the most powerful and spectacular natural phenomena [1]. It is the transfer of electrical charges between clouds or between the ground and clouds and occurs when a difference in potential of 30,000 V or higher exceeds the inherent resistance of the air [2]. Lightning strikes the earth more than 8 million times per day [3]. It is the second leading cause of weather-related death after flooding [4–6]. The risk of being struck by lightning is dependent on regional, seasonal and temporal factors [5, 7–9]. Lightning injuries peak during the summer months throughout the world [5, 7]. Most lightning accidents occur outdoors [10]. People involved in golfing, fishing, swimming, boating, camping, or hiking are particularly at risk [10].

Reviews of lightning injuries have, however, found that one-third or more occur indoors [9, 11]. As an injury mechanism, it is postulated that if lightning strikes a house, for example, the electrical energy can be transmitted through plumbing fixtures such as sinks, showers, or toilets [11]. According to a review by Lederer et al., about 10% of lightning injures occur during transport in motor vehicles [2]. Men are generally at greater risk of suffering lightning strikes than women [2, 4, 10, 12]. In reviews, a male : female ratio of 5 : 1 was found [7, 11].

Lightning strikes in humans are an uncommon but potentially devastating cause of injury [5, 13, 14]. In contrast to other high-voltage accidents, lightning-related injuries involve not only extreme high voltages, but also very short exposure times to electrical current [7, 12, 13, 15]. The injuries sustained may therefore not only be caused by electric energy, but also by high temperatures and blast waves [15]. Lightning can affect the body in six physical ways: direct strike, contact voltage, side splash, step voltage, upward streamer, and blast injury [15] (Table 2 ).

Indirect hits by lightning strike are more common than direct strikes (Table 1) [10]. A case series reported by Cherington et al. with 13 incidents found side splashes to be common [10]. Direct strikes are rare; they are responsible for about 5% of all lightning injuries and are often fatal [10, 13].

Mortality due to lightning is estimated to be 0.2–1.7 deaths per million people per year worldwide [5, 7] and is highest in Africa [5]. The incidence of lightning strikes worldwide is estimated to be 0.09–0.12/100,000 people [12]. In the UK, three people are killed annually by lightning strikes, and about 50 people are injured every year [1]. According to the Federal Statistics Agency of Switzerland, 42 people were struck by lightning between 1999 and 2009 with a mean of 3.8 per year (range 2–9) [16]. Four people were killed between 1998 and 2008 [14].

Lightning injury statistics are probably underestimated as people may not seek medical help [5, 6, 9, 12]. Also, epidemiological data is not regularly recorded and evaluated in many countries [12]. Generally, the literature about lightning strikes and related injuries is scarce [17, 18]. It consists largely of case reports and anecdotal reports [17, 18].

We analyzed lightning-related admissions to our emergency department from January 2000 to December 2010 to review and highlight their main features.

## 2. Methods

All data were collected prospectively and entered into the emergency department' centralized database (Qualicare, Switzerland) and retrospectively analyzed. The database was searched for patients aged ≥16 years admitted between 1 January 2000 and 31 December 2010.

Patients scanned for the keyword “lightning strike” (German “Blitzschlag”) were included in the analysis. Two cases with lightning strikes in their medical history that had not occurred during the study period were excluded.

Baseline demographic data (age, gender, nationality, profession) and the following clinical data were recorded: admission date, site of injury, and duration of hospital stay (<24 hours versus ≥24 hours).

## 3. Results

During the study period, 9 patients presented to our emergency room because of lightning-related injury (Table 2) with a median age of 41 years (range 24–58 years). Four were women (median age 31, range 24–32), and five were men (median age 49, range 41–58). All but one occurred on weekdays. Four people were struck outdoors: 1 on the street, 1 on a roof, and 2 in the mountains. A further 2 patients were hit while driving a car. Another patient was at home holding a copper panel when lightning struck. No site was recorded for two patients. Thus 3 of the 9 people were struck by lightning when in enclosed spaces such as a house or a car.

The most common site of injury was the nervous system (6 out of 9 patients) followed by the cardiovascular system (5 out of 9 patients). Table 2 shows details of injuries to the different organ systems. The most common neurological symptom was paresthesia which occurred in 4 cases. Two of these patients also had paralysis that resolved spontaneously. Further neurological symptoms were one case of vertigo, one of headache, and one of unreactive pupils.

Most of the cardiac symptoms were arrhythmias (4 out of 5 patients). One case of thoracic pain and one case of prehospital asystole with successful resuscitation with subsequent intermittent bradyarrhythmia were observed.

Three patients suffered skin injuries. Two patients sustained minor skin damage, and the third suffered second- and third-degree burns around the entry and exit wounds on his left chest and right-lower leg (9% body surface area) and also had first-degree burns on the whole right-lower leg. One case of deafness following lightning-associated acoustic shock was found.

Three other injuries were observed: two cases of myoglobinuria, one in relation to severe burns and one in relation to paralysis. Additionally, one case of severe muscular contraction was observed. Four of the patients had to be hospitalized for further observation. Two of the four patients were admitted to ICU.

## 4. Discussion

### 4.1. Emergency Department Experience

Our study showed men to be at greater risk of suffering a lightning strike than females. Other studies confirm these findings [2, 4, 13, 17]. Our patients were also older than the mean age in the literature, which reports that patients in their twenties and thirties have a higher risk of being struck by lightning [4, 13, 17].

Most of our patients were struck outdoors. This finding is supported by others [2, 13, 17].

Two of our patients were struck by lightning while sitting in a car. In the literature, a rate of 10% has been reported for this type of strike [2].

A review article by Slesinger et al. showed that damage to the nervous system occurs in up to 86% of cases [13]. The figures in our study were remarkably lower, and two cases of paralysis and paresthesia were observed in our study. According to a case series of 13 patients reported by Conrad, about 80% of the lightning strike victims suffered from transient paralysis and paresthesia [17], a finding we cannot confirm. The cardiovascular system was second most commonly affected by lightning strikes in our case series. Figures in a review by Slesinger et al. were slightly higher than ours with a maximum of 46% of all patients having a cardiac problem after being struck by lightning [13]. Severe burns after lightning strikes are rare [2], as we also observed in our case series. Third-degree burns do, however, occur—especially at entry and exit burns—and were present in one of our patients [18]. One of our patients suffered from deafness, which is common in lighting strike victims [13]. Two of our patients had myoglobinuria which is not unusual in lightning strikes victims [13]. Two cases of severe skeletal muscular pain were observed in our study. This may be caused by sudden skeletal muscular contractions due to electrical shock [19].

### 4.2. Risk Analysis

#### 4.2.1. Resuscitation

The immediate effects of lightning strike may cause asystole, ventricular fibrillation, or direct central nervous system injury to the respiratory center [3, 14]. According to two reviews, the latter is very important because inherent automaticity of the cardiac conduction system often restores a perfusing rhythm [5, 11]. Adequate ventilation is therefore as important as cardiac resuscitation because apnea may lead to secondary cardiac arrest [11]. According to a review by Conrad in the absence of resuscitative efforts, the duration of apnea may be a more important determinant of survival than the duration of asystole [17]. Furthermore, another review by Cherington found that lightning-related cardiac arrest occurs without any physical damage to the body in about 10% of cases [6]. They suggest that magnetic field changes associated with lightning may induce a loop current within the human torso [6]. If this loop occurs during the repolarization phase, asystole or ventricular fibrillation may occur [6]. Generally, resuscitation should be attempted on all lightning victims who appear lifeless because extraordinary recoveries even after prolonged resuscitation have been reported [3, 7, 13]. It is very important that wide unreactive pupils are not interpreted as a sign of a poor prognosis or brain death [7, 15].

#### 4.2.2. Head and Nervous System

It is difficult to evaluate the severity and consequences of damage to deep organs and tissues caused by lightning strikes [14]. Generally, the extent of injuries from electric current are dependent on voltage, electrical tissue resistance, and duration of exposure [12].

Unlike skin, nervous tissue has very low electrical resistance [2–4, 12]. According to a review by Duff and McCaffrey, nervous tissue is therefore particularly vulnerable to cell membrane damage when high voltages are applied for a short period, as with lightning strikes [4]. Damage to the cellular membranes of the nerves results in changes in cell permeability and protein denaturation which leads to cellular edema and potentially irreversible tissue damage [12]. Lightning strikes more often affect the central nervous system than the peripheral nerves [10, 11].

Furthermore, dysfunction of the autonomous nervous system may be induced, which leads to transient paralysis and paresthesia [10, 12]. According to a review by Conrad, vasospasm of small blood vessels supplying the nerves may explain these symptoms [17]. The term “keraunoparalysis” has been used to describe short-term limb weakness associated with a hyperadrenergic state after a lightning strike [10]. The symptoms usually resolve in a matter of minutes or hours [2, 10, 15]. Permanent dysfunction of the sympathetic nervous system is rare [10]. In the case series reported by Cherington et al., 80% of lightning strike victims suffered from short episodes of paralysis and paresthesia without permanent damage [10].

Further neurological findings observed involve short-term loss of consciousness, coma, seizures, headache, increased intracranial pressure, and intracranial hemorrhage [2, 11]. The nervous system is the organ system most likely to suffer complications after lightning strike [6, 10]. Delayed syndromes such as myelopathy or spinal muscular atrophy may occur weeks or even months after the lightning strike, and their exact mechanism is not known [10]. According to a review by Duff and McCaffrey, subtle cognitive impairments may be found [4].

Deafness is also common in lighting strike victims [12]. The most common cause is tympanic membrane rupture due to blast waves [1, 5, 15]. Further causes of ear injury may be barotrauma, burns, and vasomotor changes [15]. Injuries to the ear are often caused by using a telephone during a thunderstorm [9]. Acoustic injury from the loud crack of static electricity has been reported in people using a wireless phone handset with a hard-wired base during lightning [11].

#### 4.2.3. Cardiovascular System

According to a review by Lederer et al., injuries of the cardiovascular system are common in lightning strike victims [2]. It has been found that the entire myocardium is depolarized when lightning strikes and that the heart remains in forced, sustained contraction until termination of the current [2, 13]. This may cause cell necrosis, heart enzyme elevation, T-inversion, and QT prolongation similar to that reported after DC cardioversion or pacing [3]. Lightning strikes may also cause myocardial damage, pericardial effusion, conduction disturbances, and dysrhythmias [2, 3, 13]. Secondary damage to the myocardium may also occur due to catecholamine release or autonomic stimulation [2, 7]. Cardiac dysfunction, including severe biventricular failure, is often reversible [3, 13].

#### 4.2.4. Skin

Reviews show that severe burns after lightning strikes are rare [2, 7]. Zafren et al. reported that superficial current often resembles skin flashover [19]. Internal current flow leading to deep tissue necrosis is uncommon [19]. Also, the extremely short duration of exposure to electrical current (0.0001–0.003 s) may prevent deep burns [11, 12]. Zafren et al. did, however, also report third-degree burns, especially at the entry and exit points of the electrical current [19].

#### 4.2.5. Other Organ Injuries

Myoglobinuria is not an unusual finding in lighting strike victims [12]. Massive muscular contraction following electric shock may lead to release of myoglobin [12, 14], and renal failure may be precipitated by this [2, 12, 14]. According to Watanabe et al., serum creatine kinase levels are usually measured, but they never reflect the actual site and extent of the damage [14]. They may, however, be useful as a prognostic factor for the risk of acute renal failure [14].

Further injuries may be due to the sudden discharge of electrical energy causing the musculoskeletal muscles to contract forcefully, resulting in prostration or throwing the body several meters [11]. Furthermore, the rapid heating and cooling of the air around the strike causes an abrupt change in pressure, creating an explosion that may also propel the victim [11]. This may result in fractures, joint dislocation, or blunt trauma [2, 4, 12]. Falling debris such as tree trunks may also cause additional injuries [10].

#### 4.2.6. Prognosis

Zafren et al. reported that 70% of lightning strikes are not fatal [19]. They usually last only milliseconds and flash over the victim's body with minimal transfer of energy to the internal organs [13]. Lederer suggested that immediate total cessation of cellular metabolic activity following lightning strike delays the onset of anoxic degenerative processes and therefore may be responsible for the good prognosis of lightning-induced resuscitation [12]. The same mechanism may also reduce the potential for irreversible neurologic damage and may be responsible for complete neurological recovery following even prolonged resuscitation [12]. Furthermore, as the patients are mostly young, pre-arrest morbidity usually is low [5, 17].

According to Zafren et al., persons who are stunned or lose consciousness without cardiorespiratory arrest are unlikely to die [19]. Three-quarters of survivors, however, have permanent sequelae such as tinnitus, seizures, and blindness [7, 19].

#### 4.2.7. Limitations

Our study was retrospective and therefore relied on medical records which were not always complete and did not include long-term follow-up information. Only few data were available for some of our patients. Standardized data was therefore not obtained, and bias cannot be excluded. Cases presenting to our hospital may have been underreported as children are treated in a separate emergency department.

## 5. Conclusion

The patterns of clinical signs and symptoms in lightning strikes injuries are rare, are substantially different from those in common electrical accidents because extremely high voltages with brief exposure are involved, and are associated with substantial morbidity. They are therefore challenging for the attending medical team. The nervous and cardiovascular systems are especially vulnerable to damage from lightning strike. Because lightning strikes are rare, the establishment of an international register would benefit the understanding of their injury patterns and facilitate specific treatment.

## Figures and Tables

**Table 1:** Types of lightning strike and ways they affect the human body.

Type	Mechanism
Direct strike	Most of the current flows through the body; highest mortality
Contact voltage	Occurs when lightning strikes an object such as a car or metal pole that the victim is touching
Side flash	Splashing of current from a nearby object or person onto the victim
Ground current	Ground current passes from the strike point through the ground into the victim
Upward streamer	Passage of lightning from the victim upwards
Blast injury	Sudden expansive explosion of the air around the lightning channel causing blunt trauma

**Table 2:** Lightning strike victims presenting between 2000 and 2010 to our emergency department.

*N*	Year	Age	Gender	Description of event	Cardiovascular	Neurological	Skin	ENT	Other
1	03	30	Female	Patient with arrhythmias after lightning strike	Arrhythmia	/	/	/	/

2	03	45	Male	Patient was driving a car when hit by lightning, strong contraction of the pectoral muscles	Thoracic pain	/	/	/	Muscular pain

3	05	24	Female	Patient was in contact with a car when hit by lightning; short episode of paralysis and paresthesia in the right leg, first degree burns of the right hemithorax, strong pain in gluteal muscle, rhabdomyolysis, hospitalized	/	Paresthesia, paralysis	First-degree burns	/	Myoglobinuria, muscular pain

4	06	41	Male	Patient standing on the roof of a building when hit by lightning, arrhythmia, excoriation of hands, hospitalized	Arrhythmia	Vertigo	Excoriation	/	/

5	07	58	Male	Patient hiking when hit by lightning, paresthesia, first-degree burns on right-lower extremity, second- to third-degree burns on entrance point and exit point on right-lower leg and left chest, admission to ICU	/	Paresthesia	Second- to third-degree burns (9% of body surface)	/	Myoglobinuria

6	09	49	Male	Patient with arrhythmias after lightning strike	Arrhythmia	/	/	/	/

7	09	32	Female	Patient standing at a window when hit by lightning; paralysis and paresthesia of the left upper extremity for two hours	/	Paresthesia, paralysis	/	/	/

8	10	33	Female	Patient driving a car when hit by lightning; deafness of the right ear, paresthesia of the right ear, headache	/	Paresthesia, headache	/	deafness	/

9	10	52	Male	Patient working in the forest when lightning hit a tree 0.50 m away, cardiopulmonary arrest, wide nonreactive pupils, resuscitation, followed by intermittent bradyarrhythmia, admission to ICU	Asystole, arrhythmia	Unreactive pupils	/	/	/

## References

[1] Cooray V, Cooray C, Andrews CJ (2007). Lightning caused injuries in humans. *Journal of Electrostatics*.

[2] Lederer W, Wiedermann FJ, Cerchiari E, Baubin MA (2000). Electricity-associated injuries II: outdoor management of lightning- induced casualties. *Resuscitation*.

[3] Lichtenberg R, Dries D, Ward K, Marshall W, Scanlon P (1993). Cardiovascular effects of lightning strikes. *Journal of the American College of Cardiology*.

[4] Duff K, McCaffrey RJ (2001). Electrical injury and lightning injury: a review of their mechanisms and neuropsychological, psychiatric, and neurological sequelae. *Neuropsychology Review*.

[5] Ritenour AE, Morton MJ, McManus JG, Barillo DJ, Cancio LC (2008). Lightning injury: a review. *Burns*.

[6] Cherington M (2003). Neurologic manifestations of lightning strikes. *Neurology*.

[7] Hinkelbein JSO, Wetsch WA Blitzschlag und Blitzunfälle in der präklinischen Notfallmedizin: Relevanz, Folgen und praktische Implikationen.

[8] (2000). Part 8: advanced challenges in resuscitation. Section 3: special challenges in ECC. 3G: electric shock and lightning strikes. European Resuscitation Council. *Resuscitation*.

[9] Elsom DM (2000). Deaths and injuries caused by lightning in the United Kingdom: analyses of two databases. *Atmospheric Research*.

[10] Cherington M, Yarnell PR, London SF (1995). Neurologic complications of lightning injuries. *Western Journal of Medicine*.

[11] Mistovich JJ, Krost WS, Limmer DD (2008). Beyond the basics: lightning-strike injuries. *EMS Magazine*.

[12] Lederer WKG (2005). Notfallmedizinische Versorgung von Blitz- und Stromschlagverletzungen. *Anaesthesist*.

[13] Slesinger TL, Bank M, Drumheller BC (2010). Immediate cardiac arrest and subsequent development of cardiogenic shock caused by lightning strike. *Journal of Trauma*.

[14] Watanabe N, Inaoka T, Shuke N (2007). Acute rhabdomyolysis of the soleus muscle induced by a lightning strike: magnetic resonance and scintigraphic findings. *Skeletal Radiology*.

[15] Fred Z (2007). Blitzunfall-Energieübertragungsmechanismen und medizinische Folgen. *Deutsches Ärzteblatt*.

[16] BSF http://www.bfs.admin.ch/bfs/portal/de/index/themen/14/04/01/data/01/01.html.

[17] Conrad L (1998). Clinical update on lightning injuries. *Wilderness and Environmental Medicine*.

[18] Muehlberger T, Vogt PM, Munster AM (2001). The long-term consequences of lightning injuries. *Burns*.

[19] Zafren K, Durrer B, Herry JP, Brugger H (2005). Lightning injuries: prevention and on-site treatment in mountains and remote areas: official guidelines of the International Commission for Mountain Emergency Medicine and the Medical Commission of the International Mountaineering and Climbing Federation (ICAR and UIAA MEDCOM). *Resuscitation*.

